# Vaccine-related poliovirus shedding in trivalent polio vaccine and human immunodeficiency virus status: analysis from under five children

**DOI:** 10.1186/s13104-017-2843-y

**Published:** 2017-11-03

**Authors:** Joanne Hassan, Laura Wangai, Peter Borus, Christopher Khayeka–Wandabwa, Lucy Wanja Karani, Mercy Kithinji, Michael Kiptoo

**Affiliations:** 1 Institute of Tropical Medicine and Infectious Diseases (ITROMID), Jomo Kenyatta University of Agriculture and Technology (JKUAT), Juja, Kenya; 2 Kenya Medical Research Institute (KEMRI), P.O. Box 54628-00200, Nairobi, Kenya; 3 Kirinyaga University, Kerugoya, Kenya; 4 World Health Organization (WHO), Nairobi, Kenya; 5 Health Sciences Platform, School of Pharmaceutical Science and Technology (SPST), Tianjin University, 92 Weijin road, Nankai District, Tianjin, 300072 People’s Republic of China; 6 African Population and Health Research Center (APHRC), Nairobi, Kenya; 7 Cottolengo Children’s Home, Nairobi, Kenya; 8 South Eastern Kenya University (SEKU), Kwa Vonza, Kenya

**Keywords:** Poliomyelitis, Poliovirus, Poliovirus shedding, HIV, Immunization, Vaccination, Real time polymerase chain reaction

## Abstract

**Background:**

Poliomyelitis is an acute viral infection caused by poliovirus and transmitted via the fecal–oral route. The causative agent is one of the three serotypes of poliovirus (serotypes 1, 2, 3) that differ slightly in capsid protein. Prolonged vaccine-related poliovirus shedding in human immunodeficiency virus (HIV) positive individuals has been linked to possible reservoir for reintroduction of polioviruses after eradication. The study therefore aimed at estimating the duration for vaccine-related poliovirus shedding among potentially and HIV-infected persons.

**Methods:**

Poliovirus excretion was studied following vaccination of children aged ≤ 59 month per human immunodeficiency virus status after national immunization days. Their medical records were reviewed to identify the child’s HIV status, demographic and immunization data. Sequential stool samples were collected at site 2nd, 4th and 8th week after trivalent oral poliovirus vaccine (tOPV) was administered. To isolate suspected polioviruses and non-polio enteroviruses, characterize poliovirus subtypes by intratypic differentiation and Sabin vaccine derived poliovirus, real time polymerase chain reaction was applied. Shedding for ≥ 24 weeks was defined as long-term persistence.

**Results:**

The mean age of the study population was 28.6 months, while the median age was 24 months. Of the children recruited, majority were in the 25–48 months (n = 12; 46.2%) age category. All the HIV-positive children (n = 10) had mild symptomatic HIV status and did shed vaccine-related polioviruses between weeks 2 and 4 respectively. No participant shed polioviruses for ≥ 6 weeks.

**Conclusions:**

It was evident mildly symptomatic HIV+ children sustain the capacity to clear vaccine-related poliovirus. The oral poliovirus vaccine-2 (Sabin like) that was detected in one HIV-infected child’s stool 6 weeks after the national immunization days was predominantly non revertant. There was no evident prolonged poliovirus shedding among the participants enlisted in the present study. High powered studies are desired to further corroborate these findings.

## Background

After polio eradication, it is broadly advanced that paralytic poliomyelitis will mainly occur as a consequence of sustained use of live oral poliovirus vaccine (OPV) [1–6]. Vaccination policies on the post-eradication era discussions are underway with progress toward eradication of poliovirus (PV) circulation in most regions and countries globally [1, 7]. After the global eradication of polioviruses the potential of persistence and circulation vaccine-derived polioviruses (VDPV) is a risks that cannot be underestimated when advancing strategies towards stopping polio vaccination [7].

According to World Health Organization (WHO) OPV is considered safe and immunogenic in HIV-infected persons [8, 9] and is widely used as a primary approach for global polio eradication initiative for instance in Africa where about 90% of the two million children with HIV live [10]. In this population segment of HIV positive children few cases of vaccine-associated paralytic poliomyelitis (VAPP) have earlier been reported [11, 12]. In consideration of the high proportions of potentially and HIV positive persons in developing countries eligible for and therefore exposed to OPV [9, 13], and the public health implications of possible prolonged VDPV persistence, the need to evaluate and appraise the risk progressively should be emphasized. Genetically, VDPVs have been shown to have > 1% sequence difference from the Sabin vaccine virus [14]. Mutations of OPV can lead to a form that can achieve neurological infection and consequently cause paralysis [15] this neurological infection include paralysis caused by VDPV and is indistinct from that caused by wild polioviruses by means of sequencing [16]. However, this is a rare occurrence with the rate of vaccine-associated paralytic poliomyelitis (VAPP) varying by region and at about 1 case per 750,000 vaccine recipients [17]. Reported outbreaks of VAPP tend to happen in areas of low OPV coverage and more likely to occur in adults than in children with pre-assumption being OPV is itself protective against the related outbreak strain [4, 18, 19].

Further circulation risk of poliovirus in ineffectively immunized persons arises when immunocompromised individuals with persistent VDPV may serve as a latent reservoir for possible reintroduction of PV into the overall population following wild poliovirus elimination. HIV infection during perinatal phase is correlated with humoral and cellular immunodeficiencies. It has been advanced that perinatally HIV infected children have been identified to have a polyclonal hypergammaglobulinemia paradoxically linked with reduced antibody responses to specific antigens [20–22]. Furthermore, individuals with certain defects of antibody production are known to be affected by chronic VDPV persistence [1, 23], with limited insights about PV persistence among HIV+ persons. In associated studies conducted, a case of enterovirus excretion was noted in an HIV+ child for up to 6 months [24, 25] and related findings pointed to the concern that, HIV-positive children are more prone to enteroviruses as compared to their healthy counterparts [24, 25]. Further findings have pointed to varied observations of non-severe or severe enterovirus infection among HIV-infected individuals [26–28]. Available findings do not support the increased risk and severity of wild or vaccine-associated poliomyelitis in HIV-infected individuals. Immunization using OPV of HIV-infected children results in protective, although rather lower, antibody titres as compared with uninfected children, with a comparable low rate of adverse events across the groups [29, 30]. In this regard, whether children perinatally infected with HIV may be source of VDPV is a query yet to be fully explored. In order to provide more insights, the study aimed to examine vaccine derived poliovirus shedding among potentially and HIV positive children who are under 5 years of age to asses if there is prolonged virus replication and shedding that would eventually serve as a reservoir for paralytic poliovirus. We conducted a prospective cross sectional study evaluating stool shedding of vaccine strains and polio seroconversion in Kenyan children who received OPV per the national immunization schedule. The highly desired incremental evidence [31–33] could additionally benefit the global and regional policy environment on shaping the discourse around public health implications of potential VDPV, inform the potential need for large scale rigorous studies and the contention on the risk of the VDPV persistence among HIV-infected persons [4, 7, 19, 31].

## Methods

### Study population and specimen collection

This was a prospective cross sectional study conducted at Cottolengo children’s orphanage home in Nairobi, Kenya from August 2013 until June 2014. The orphanage has a capacity of 500 children and admits mainly orphaned children between birth and 18 years whose parents have died of HIV related complications. The institution accords the children with adequate nutritional and medical care inclusive of comprehensive immunization. The reliable operational scope of the children’s home alongside its progressively appraised medical records and tracking systems with demographic, clinical and immunization database, provided a rigorous and dependable platform for the prospective study. In addition, prior experience with similar target population reliability in closely related settings has been proven [10, 34]. For inclusion into the study, stepwise considerations and parameters were followed as elaborated. All children enrolled in the study were below the age of 5 years. Medical records at the orphanage were reviewed to identify each child’s HIV status and obtain age, sex, and immunization data. Final HIV infection status of the participants was assigned at the time of sample collection in September 2013. One blood vial sample for confirmatory HIV status and CD4+ count per national screening protocol [National AIDS and STI Control Programme (NASCOP)] was collected in August 2013 just before NIDs (effective September), Children whose status had reverted to seronegative were categorized as HIV-uninfected and assigned to the control group. All enrolled infants received trivalent oral poliovirus vaccine (tOPV) during the September NIDs. Serial stool specimens collection at site was done during the 2nd, 4th, 6th and 8th week after vaccination with tOPV. The last OPV exposure dates were defined as the date of the national immunization days (NIDs) for all children ≤ 59 months. Severely ill children were excluded. Informed consent of legal foster parents/tutors was obtained before inclusion in the study. Thus, infants were included in the study if (1) their age was ≤ 59 months at the time of enrollment (2) had their HIV status confirmed (3) their OPV status history could be verified, and (4) they had available stool. The entire stool samples from HIV-infected and uninfected children were processed in stool-shedding analysis. The data collection processes were performed by trained clinical nurse and laboratory technologist on the methodology and objectives in coordination by investigators JH and MK1 at the facility. All samples were processed at Kenya Medical Research Institute Nairobi (KEMRI).

#### Laboratory analysis determination of HIV-1 infection status and T cell subset analysis

The whole blood samples were collected in an ethylenediaminetetraacetic acid-containing Vacutainer (purple-top), transported on ice to the Centre for virus research HIV laboratory at Kenya Medical Research Institute (KEMRI) for analysis. HIV status was determined by HIV PCR using an Amplicor HIV DNA Test Version 1.5 kit and a GeneAmp 9700 thermal cycler. HIV enzyme-linked immunosorbent assay (ELISA) was performed to confirm HIV status at 18 months of age. Confirmation was defined as ≥ 2 positive HIV tests with ≥ 1 done as part of the study, or ≥ 1 positive HIV test done as part of the study and clinical depiction strongly suggestive of HIV infection. In line with their HIV status, the children were classified into two groups: (i) HIV-infected (n = 10), and (ii) HIV-uninfected (n = 16). CD4 T-cell count was carried out on the HIV positive using a FACS Calibur Flow Cytometer (Becton-Dickinson Immunocytometry Systems, San Jose, CA, USA), as per the manufacturer’s instructions [35, 36].

#### Virus isolation and identification

Stool specimens were transported on frozen ice packs to the Centre for virus research Poliovirus laboratory at Kenya Medical Research Institute (which is also a WHO National lab in the AFRO region). Virologic isolation and identification was done according to WHO Polio Laboratory Network Standard Protocols (Polio laboratory manual 4th edition, 2004). Briefly, stool extracts were inoculated in RD (human rhabdomyosarcoma derived cells) and murine L20B (a transgenic mouse L cells) cell lines. The L20B cell line was used to distinguish polioviruses from non-polio enteroviruses because the mouse cell lines is genetically modified to express human cellular receptor for polioviruses allowing selective poliovirus culture. Positive RD cell cultures were passaged in L20B to distinct poliovirus and non-polio enteroviruses. Positive L20B isolates were harvested for PCR.

### Real time reverse transcriptase-polymerase chain reaction (rRT-PCR) for intratypic differentiation (ITD) and Sabin VDPV

The poliovirus diagnostic real -time RT PCR was performed on L20B positive cell cultures that were suspected to contain poliovirus. Briefly, the viral RNA (vRNA) was converted to complementary DNA (cDNA) using reverse transcriptase. The cDNA was amplified in a PCR reaction using Taq polymerase. The PCR products were detected and identified by hybridization with specific Taqman^®^ probes. Both the cDNA synthesis and the PCR reaction used multiple sets of oligonucleotide primers that were tagged with probes with different specificities. This combination of primers and probes resulted in the serotype identification and intratypic differentiation of poliovirus isolates (as described in World Health Organization, Polio Laboratory Manual 4 ed. 2004, Geneva.). Using an ABI 7500 system the run conditions used were as follows: 95 °C for 24 s, 44 °C for 30 s, then a 25% ramp speed to 60 °C for 24 s, for 40 cycles. The primers (manufactured by Center for Disease Control and Prevention, USA) targeting the VP1 gene ~ 900 nucleotides were utilized to determine if the sample contained polioviruses or non-polio enterovirus. The type of poliovirus was determined as wild type or vaccine derived (sabin) using primer specific for wild and VDPV. This was done using the detailed primer sequence in Table 1.

**Table 1:** Primer or probe sequence for intratypic differentiation (ITD)

Specificity	Primer or probe (polarity)	Primer or probe sequence (5′ → 3′)
Pan-enterovirus	PCR-1(A) PCR-2 (S) PanEV probe (S)	GCGATTGTCACCATWAGCAGYCA GGCCCCTGAATGCGGCTAATCC **FAM**-CCGACTACTTTGGGWGTCCGTGT-**BHQ1**
Pan-poliovirus^a^	panPV/PCR-1(A) panPV/PCR-2(S) panPV Probe21A(A)	AYRTACATIATYTGRTAIAC CITAITCIMGITTYGAYATG **FAM**-TGRTTNARIGCRTGICCRTTRTT-**BHQ1**
Serotype 1^a^	seroPV1A(A) seroPV1,2S(S) seroPV1 probe 16A(A)	ATCATIYTPTCIARPATYTG TGCGIGAYACIACICAYAT **FAM**-TGCCYAVICCYGIGMIADYGC-**BHQ1**
Serotype 2	seroPV2A (A) seroPV1,2S (S) seroPV2,probe 5S (S)	AYICCYTCIACIRCICCYTC TGCGIGAYACIACICAYAT **FAM**-CARGARGCIATGCCCICARGGIATNGG-**BHQ1**
Serotype 3^a^	seroPV3A (A) sero PV 3S (S) seroPV3,probe 11S (S)	CCCIAIPTGRTCRTTIKPRTC AAYCCITCIRTITTYTAYAC **FAM**-CCRTAYGTNGGITTRGCVAAYGC-**BHQ1**
Sabin 1	Sab1/PCR-1(A) Sab1/PCR-2(S) Sab1/probe (A)	CCACTGGCTTCAGTGTTT AGGTCAGATGCTTGAAAGC **CY5**-TTGCCGCCCCCACCGTTTCACGGA-**BHQ3**
Sabin 2	Sab2/PCR-1(A) Sab2/PCR-2(S) Sab2/probe (S)	CGGCTTTGTGTCAGGCA CCGTTGAAGGGATTACTAAA **FAM**-ATTGGTTCCCCCGACTTCCACCAAT-**BHQ1**
Sabin 3	Sab3/PCR-1(A) Sab3/PCR-2(S) Sab3/probe (S)	TTAGTATCAGGTAAGCTATC AGGGCGCCCTAACTTT **ROX**-TCACTCCCGAAGCAACAG-**BHQ2**

Degenerate primers: K=G and T; M=A and C; R=A and G; Y=C and T; I *=* degenerate base analog, Inosine; P = degenerate base for (TC)

^a^We used degenerate PCR conditions with these primer sets

The vaccine derived poliovirus samples were further tested for the degree of relatedness to prototype Sabin strains per standard procedure from the kit manufacturer (Center for Disease Control and Prevention, USA). The primer sequence used for the real time PCR was as shown in Table 2.

**Table 2:** Primer or probe sequence for Sabin vaccine derived poliovirus (VDPV)

Primer specificity	Primer and probe sequences 5′ → 3′
S1 VDPV VP1 (Target aa# 99)	Sense CATGCGTGGCCATTATA Anti-sense CAAATTCCATATCAAATCTA VP1 probe **FAM**-CACCAAGAATAAGGATAAGC-**BHQ1**
S2 VDPV VP1 (Target aa# 143)	Sense GACATGGAGTTCACTTTTG Anti-sense CTCCGGGTGGTATATAC VP1 probe **FAM**-CATTGATGCAAATAAC-**BHQ1**
S3 VDPV VP1 (Target aa# 285–290)	Sense CATTTACATGAAACCCAAAC Anti-sense TGGTCAAACCTTTCTCAGA VP1 probe **FAM**-TAGGAACAACTTGGAC-**BHQ1**

### Statistical analysis

Data were managed using excel spread sheet and Statistical Package for Social Sciences (SPSS) version 21. Quantitative data was analyzed by means of descriptive statistics. Parameters considered in estimating the proportions shedding vaccine poliovirus serotypes entailed time from the last OPV dose administered, HIV status, PV seroconversion, and age. Immuno competence was defined per the WHO/CDC age specific clinical categories and immunologic status: (1) no evidence of immunosuppression—age < 12 months: CD4+ count ≥ 1500, and/or CD4% ≥ 25%; age 1–5 years: CD4+ count ≥ 1000 and/or CD4+ % ≥ 25%; (2) moderate immunosuppression—age < 12 months: CD4+ count 750–1499, and/or CD4% 15–24%; age 1–5 years: CD4+ count 500–999 and/or CD4+ % 15–24%; (3) severe immunosuppression—age < 12 months: CD4+ count < 750, and/or CD4% < 15%; age 1–5 years: CD4+ count < 500 and/or CD4+ % 15% [36, 37]. In the rRT-PCR for ITD and VDPV, primers that specifically differentiate between wild and vaccine derived polio were utilized. Pearson’s Chi squared test was applied to determine whether there was a significant difference in the distribution of PV serotypes between the HIV+ and HIV− groups. P values of equal or less than 0.05 (*P* ≤ 0.05) were considered to be statistically significant.

## Results

The study recruited 26 children ≤ 59 months from the home. On profiling the children HIV status as at September 2013 (the time when national immunization activities was done), 10 were confirmed to be HIV-infected, whereas the rest (16 children) had sero-negative status and were therefore categorized as HIV-uninfected. The mean of CD4 count among the HIV infected children was 1450/mm^3^ confirming no child was considered immunodepressed among the enrolled despite having the virus and factoring the age range of those enrolled [37, 38]. The mean age of the study population was 28.6 months, while the median age was 24 months. Of the children recruited, majority were in the 25–48 months (n = 12; 46.2%) age category followed by 13–24 months age category (n = 8; 30.8%). There were slightly more females (n = 14; 53.8%) recruited than males (n = 12; 46.2%). The demographic characteristics of the study subjects are as elaborated in Table 3. The mean age of the HIV positive study population was 32.4 months, while the median age was 30 months; the mean age of the male study participants was 29.1 months with a median of 24 months whereas the mean age of corresponding females was 40 months with a median of 36 months. For the HIV negative study population the mean age was 26.2 months with a median of 27 months; the mean age of the male study participants was 28.6 months while the females was 25.3 months with a median age of 30 and 24 months for male and female in that order. Medical records review showed that only one child had been ill during the immunization rounds period which could have been a contraindication to supplemental doses of OPV during NIDs.

**Table 3:** The demographic characteristics of the study subjects

Characteristics of the HIV positive and negative (control) study subjects (N = 26)		
	Frequency of N	Percentage (%)
Age (months)		
< 12	4	15.4
13–24	8	30.8
25–48	12	46.2
49–60	2	7.7
Sex		
Female	14	53.8
Male	12	46.2
HIV status		
Positive	10	38.5
Negative	16	61.5

Characteristics of the HIV positive study subjects, CD4 count mean and ranges (n = 10)		
	Frequency of n	Percentage (%)
Age (months)		
< 12	1	10
13–24	2	20
25–48	5	50
49–60	2	20
Sex		
Female	3	30
Male	7	70

Sex	Mean CD4 count (mm^3^)	CD4 range (mm^3^)
Male	1302	1000–1900
Female	1795	1600–2000

Characteristics of the HIV negative (control) study subjects (n = 16)		
	Frequency of n	Percentage (%)
Age (months)		
< 12	3	18.7
13–24	6	37.5
25–48	7	43.8
Sex		
Female	11	68.8
Male	5	31.2

Findings of poliovirus isolation in stool specimens by HIV serostatus are as presented in Table 4. A total of 80 stool specimens were obtained during the 8 week study period, 31 from HIV-infected children and 49 from HIV-uninfected children. Of the collected specimens 53 were positive for suspected poliovirus, 23 from HIV-infected children and 30 from HIV-uninfected children. There was a positive viral culture results at some point for each child during the study period.

**Table 4:** Virus isolation verses HIV status of study subjects

Sample size	Total, n	HIV(+), n	HIV(−), n
Children in the study	26	10	16
Isolation rate for suspected polioviruses and NPEV			
Specimens with suspected polioviruses or NPEV isolated in week 2	26	10	16
Specimens with suspected polioviruses or NPEV isolated in week 4	26	10	16
Specimens with suspected polioviruses or NPEV isolated in week 6	1	1	0
Specimens with suspected polioviruses or NPEV isolated in week 8^a^	1	0	1

^a^One study subject who was HIV-uninfected shed NPEV up to week 8 of sample analysis

On poliovirus shedding duration, there was no observable evidence of prolonged PV or non-polio enteroviruses (NPEV) shedding with no PV detected in any of the specimens at 8 weeks after the last OPV dose. It was evident; all PV isolates from the enrolled study participants were vaccine-related. At some point of the study each study subject shed one form or another of the vaccine derived poliovirus as seen on Table 5. Type 1 poliovirus was detected in 16 specimens (three specimens from HIV-infected children), type 2 in three specimens 2 of which were from HIV-infected children and type 3 in 20 specimens (six from HIV-infected). More than one poliovirus type was identified in 14 specimens of which six were from HIV-infected children. More than one specimen among the 26 children (10 HIV-infected and 16 HIV-uninfected) was positive for poliovirus. There was no observable significant difference in the distribution of PV serotypes between the HIV positive and HIV negative groups with the virus shedding pattern between the groups in the order showing no significant difference as well (*P* > 0.05) by sex or age. Among the study participants (Table 6), poliovirus serotype 3 was shed by majority of the children (38.5%) in week 2 and week 4 respectively with the highest shedding among ages 13–24 months (15.4 and 19.2%) in week 2 and 4 in the order. However, poliovirus serotype 1 was also shed most by ages 25–48 months (15.4%). There was no evidence from our data that age of the study participants was a contributing factor to prolonged (≥ 6 months) poliovirus shedding.

**Table 5:** Duration of shedding vaccine after immunization verses the proportional study subjects at the time points and HIV status

Parameters (time and poliovirus)	Total, N = 26, (%)	HIV(+), n = 10, (%)	HIV(−), n = 16, (%)
2 weeks			
PV 1	9 (34.6)	3 (30)	6 (37.5)
PV 2	1 (3.8)	1 (10)	0 (0)
PV 3	10 (38.5)	4 (40)	6 (37.5)
PV 1 + 2	1 (3.8)	1 (10)	0 (0)
PV 1 + 3	3 (11.5)	1 (10)	2 (12.5)
PV 1 + 2 + 3	1 (3.8)	0 (0)	1 (6.3)
PV 1 + 2 + 3 + NPEV	1 (3.8)	0 (0)	1 (6.3)
4 weeks			
PV 1	7 (26.9)	2 (20)	5 (31.3)
PV 2	1 (3.8)	1 (10)	0 (0)
PV 3	10 (38.5)	3 (30)	7 (43.8)
PV 1 + 2	1 (3.8)	1 (10)	0 (0)
PV 1 + 3	5 (19.2)	3 (30)	2 (12.5)
PV 2 + 3	1 (3.8)	0 (0)	1 (6.3)
PV 1 + 2 + 3	1 (3.8)	0 (0)	1 (6.3)
PV 1 + 3 + NPEV	1 (3.8)	0 (0)	1 (6.3)
6 weeks			
Negative	24 (92.3)	9 (90)	15 (93.8)
PV 1	0 (0)	0 (0)	0 (0)
PV 2	1 (3.8)	1 (10)	0 (0)
NPEV	1 (3.8)	0 (0)	1 (6.3)
8 weeks			
Negative	25 (96.2)	10 (100)	15 (93.8)
NPEV	1 (3.8)	0 (0)	1 (6.3)

**Table 6:** Duration and proportions of participants shedding vaccine after immunization verses the age of the study subjects

Parameters	Total, n = 26 (%)	< 12 months	13–24 months	25–48 months	49–60 months
2 weeks					
PV 1	9 (34.6)	1 (3.8)	2 (7.7)	4 (15.4)	2 (7.7)
PV 2	1 (3.8)	0 (0)	0 (0)	1 (3.8)	0 (0)
PV 3	10 (38.5)	3 (11.5)	4 (15.4)	3 (11.5)	0 (0)
PV 1 + 2	1 (3.8)	0 (0)	0 (0)	1 (3.8)	0 (0)
PV 1 + 3	3 (11.5)	0 (0)	2 (7.7)	1 (3.8)	0 (0)
PV 1 + 2+3	1 (3.8)	0 (0)	0 (0)	1 (3.8)	0 (0)
PV 1 + 2 + 3 + NPEV	1 (3.8)	0 (0)	0 (0)	1 (3.8)	0 (0)
4 weeks					
PV 1	7 (26.9)	2 (7.7)	1 (3.8)	3 (11.5)	1 (3.8)
PV 2	1 (3.8)	0 (0)	0 (0)	1 (3.8)	0 (0)
PV 3	10 (38.5)	2 (7.7)	5 (19.2)	3 (11.5)	0 (0)
PV 1 + 2	1 (3.8)	0 (0)	0 (0)	0 (0)	1 (3.8)
PV 1 + 3	5 (19.2)	0 (0)	2 (7.7)	3 (11.5)	0 (0)
PV 2 + 3	1 (3.8)	0 (0)	0 (0)	1 (3.8)	0 (0)
PV 1 + 2 + 3	1 (3.8)	0 (0)	0 (0)	1 (3.8)	0 (0)
PV 1 + 3 + NPEV	1 (3.8)	0 (0)	0 (0)	1 (3.8)	0 (0)
6 weeks					
Negative	24 (92.3)	4 (15.4)	8 (30.8)	10 (38.5)	2 (7.7)
PV 2	1 (3.8)	0 (0)	0 (0)	1 (3.8)	0 (0)
NPEV	1 (3.8)	0 (0)	0 (0)	1 (3.8)	0 (0)
8 weeks					
Negative	25 (96.2)	4 (15.4)	8 (30.8)	11 (42.3)	2 (7.7)
NPEV	1 (3.8)	0 (0)	0 (0)	1 (3.8)	0 (0)

In the genetic characterization of PV isolates, the VP1 gene was the gene of interest for the isolates. Poliovirus isolates appeared “Sabin-like” in rRT PCR intratypic differentiation tests, indicating the lack of any major antigenic changes. All the poliovirus isolates that tested positive during the intratypic differentiation (ITD) for any Sabin-like polioviruses (*n* = 53) from the 26 study subjects on week 2–6 were tested for possible recombination by real-time PCR; all did amplify with the VP1 genomic region using the homotypic Sabin-specific primers, ruling out the evidence of recombination with other circulating vaccine-related or wild polioviruses. One HIV-infected participant shed a combination of poliovirus type 2 sabin like and non polio enterovirus (NPEV) up to week 6, but continued shedding NPEV up to week 8.

## Discussion

Oral poliovirus vaccine has been a vaccine of choice in developing countries for both persons infected with HIV and the uninfected. Previous studies in Democratic Republic of Congo showed high protective antibody titres in HIV infected children who had received the three OPV doses [29, 30]. Shedding of poliovirus in immunized persons is usually between 2 and 3 months [29, 30, 39]. We evaluated vaccine poliovirus shedding duration in stool of HIV positive and HIV negative Kenyan children who received OPV per the National immunization activities schedule. We observed that although prolonged poliovirus shedding was not associated with the children’s HIV status, multiple serotypes shedding was noted in all study subjects in 2nd and 4th weeks respectively. In resonating Kenyan findings, to evaluate the potential for VDPV persistence among HIV-infected persons, a proportion of HIV seropositive children enrolled was confirmed to be HIV-infected. There was no clinical indication of prolonged persistence of vaccine-related polioviruses or NPEV. Further, in all cases (infected and uninfected), the poliovirus infections were cleared with the overall trends of excretion not differing by HIV status. The pointed phenomenon of non-variation in excretion by HIV or non-HIV status have also been noted in a longitudinal outcomes [10]. Subsequent to vaccination with OPV there is decreased Poliovirus shedding which is broadly considered a marker for the development of mucosal intestinal immunity [40]. In the present study, there was a decrease in poliovirus shedding in weeks 6 and 8 suggesting that both HIV infected and uninfected children developed intestinal mucosal immunity after OPV dose. Age was also not a factor that affected the pattern or duration of OPV shedding. Prior reporting has observed that HIV-infected children shed vaccine-related polioviruses in between 15 and 42 months based on specimens obtained after the last known OPV dose consequently suggesting possible long-term persistence [41]. However, further molecular findings pointed to the fact that for all three cases, there was a variance of nucleotide sequences in the VP1 region of 0.3–0.6% showing close genetic relatedness to prototype Sabin strains. Thus, it was worth estimating the frequency of such individuals in countries with varying socioeconomic and health policies context for poliomyelitis control. In our presented findings though in a shorter time period but more participants, affirms the ability to clear OPV is preserved across HIV-infected and uninfected children.

A range of factors could possibly influence the course of poliovirus infection among the HIV-infected and these include the degree of immunosuppression, previous OPV vaccination history, and patient age. The CD4 lymphocyte count of HIV-infected individuals is a significant predictor of HIV progression as well as acquired immune deficiency syndrome (AIDS)-associated mortality [30]. In the present study, following HIV status evaluation, we considered CD4 count to assess immune competence and there after adopted the “gold standard” for assessing mucosal immunity after vaccination with PV vaccines which consists of measuring virus excretion in stool after challenge with OPV. The absence of or reduced shedding is an indicator of mucosal intestinal protection. Further, immune protection to poliomyelitis is known to prevail in two forms—humoral and mucosal where humoral immunity protects from paralytic poliomyelitis and protection against disease correlates with induction of serum poliovirus-neutralizing antibody [42, 43]. On the contrary, mucosal immunity is presumed to protect against poliovirus entry into and transmission from the intestinal and nasopharyngeal mucosae which is the primary sites of poliovirus replication. The clinical categorization and immunologic profiles of HIV positive children was assigned according to the 1994 Centers for Disease Control and Prevention (CDC) revised classification which is age specific defined immunologic status [36]. Considering HIV infection is mainly a mucosal disease and the gastrointestinal tract (GIT) is the foremost site for the virus replication given that it houses most of the body’s lymphocytes (including activated memory CD4+ T cells that are preferential targets for HIV), infection and progression of HIV is mainly characterized by incessant activation, rapid turnover, and activation-induced cell death of CD4+ and CD8+ T cell populations [44, 45]. The degree of immune activation represents an independent and more powerful predictor of CD4+ T cell homeostasis, immune and disease progression [46, 47]. Thus we did not directly target mucosal immunity parameters due to associated determinants [48] nonetheless, progressive validation of mucosal immunity markers is desired depended on how beneficial they will be in relation to transition from using OPV to IPV [40, 48]. Overall mean CD4 counts established in the HIV infected children was not characteristic to progression of disease and the PCR analysis of viral protein 1 (VP1) region of the Sabin like isolates showed no difference with the one of the vaccine administered therefore there was no reversion to neurovirulence.

However, this study had some limitations, other than the small sample size, the present study population possibly present a segment of vulnerable population well catered for within the orphanage in terms of any arising medical and nutritional attention needs hence enhanced immune competence. Thus, the participants segment may not be completely representative of the typical general population of HIV-infected children in Kenya and in other developing countries where nutrition support and cultural barriers would be a challenge to effective HIV status management and hence possible response to OPV.

Cognizant of the fact that about 90% of the two million HIV positive children live in Africa [10], the presented finding shed insights into context specific setting results that can be the basis for comparison with other pockets of findings in the country and sub-Saharan region to information and/or formulation of a basis for a possible population based and more rigorous representative national or regional evidence for policy relevant findings in the pan African context. This is in appreciation and consideration of the fact that progress towards eradication of poliovirus globally is more tenable than ever before and discussions are underway on workable and context resilient vaccination policies in the post-eradication era [1, 40, 49]. Thus, potential or possible large scale research evidence anchored on and/or informed by pockets of preliminary small scale observations, will be core in streamlining evidence based policy and operational guidelines for success of any vaccination programs post eradication of polio and in the overall continuum of healthcare.

## Conclusion

In consideration of the scope of the presented findings, it is indicative that HIV+ children retain the capacity to clear vaccine-related PV. The OPV-2 (Sabin Like) that was detected in one HIV-infected child’s stool 6 weeks after the NID was predominantly non-revertant. Elaborate longitudinal and high powered studies covering full range of HIV disease and robust follow up period are needed to fully shed more insights on the patterns of VDPV excretion that can potentially lead to revertant poliovirus among HIV+ persons and further quantify the risk magnitude of long-term VDPV replication in this group.

## Abbreviations

PV: poliovirus
tOPV: trivalent oral poliovirus vaccine
NPEV: non-polio enteroviruses
ITD: intratypic differentiation
VDPV: vaccine derived poliovirus
PCR: polymerase chain reaction
VAPP: vaccine-associated paralytic poliomyelitis
VDPV: vaccine-derived polioviruses
VP1: viral protein 1
HIV: human immunodeficiency virus
NIDs: national immunization days
NASCOP: National AIDS and STI Control Programme
WHO: World Health Organization
rRT-PCR: real time reverse transcriptase- polymerase chain reaction
cDNA: complementary deoxyribonucleic acid
ITD: intratypic differentiation
